# InstructPLM-mu: protein fitness prediction from structure instructions

**DOI:** 10.1093/bib/bbag400

**Published:** 2026-10-09

**Authors:** Junde Xu, Yapin Shi, Lijun Lang, Taoyong Cui, Zhiming Zhang, Guangyong Chen, Jiezhong Qiu, Pheng-Ann Heng

**Affiliations:** Department of Computer Science and Engineering, The Chinese University of Hong Kong, Shatin, New Territory, Hong Kong SAR, 999077, China; Hangzhou Institute for Advanced Study, University of Chinese Academy of Sciences, No. 1, Xiangshan Zhinong, Xihu District, Hangzhou, Zhejiang, 310024, China; Department of Computer Science and Engineering, The Chinese University of Hong Kong, Shatin, New Territory, Hong Kong SAR, 999077, China; Department of Computer Science and Engineering, The Chinese University of Hong Kong, Shatin, New Territory, Hong Kong SAR, 999077, China; Hangzhou Institute of Medicine, Chinese Academy of Sciences, 150 Dongfang Street Xiasha, Qiantang District, Hangzhou, Zhejiang, 310000, China; College of Intelligence and Computing, Tianjin University, No. 135 Yaguan Road, Haihe Education Park, Tianjin, 300350, China; Hangzhou Institute of Medicine, Chinese Academy of Sciences, 150 Dongfang Street Xiasha, Qiantang District, Hangzhou, Zhejiang, 310000, China; Hangzhou Institute of Medicine, Chinese Academy of Sciences, 150 Dongfang Street Xiasha, Qiantang District, Hangzhou, Zhejiang, 310000, China; Department of Computer Science and Engineering, The Chinese University of Hong Kong, Shatin, New Territory, Hong Kong SAR, 999077, China

**Keywords:** protein language models, multimodal fusion, mutation prediction, fine-tuning

## Abstract

Multimodal protein language models (PLMs) deliver strong performance on mutation-effect prediction, but training such models from scratch demands substantial computational resources. In this paper, we propose a fine-tuning framework called InstructPLM-mu and investigate the question: Can a pretrained sequence-only PLM, augmented with structural information during fine-tuning, match the performance of end-to-end trained multimodal models? Surprisingly, our experiments show that fine-tuning ESM2 with structural inputs can reach performance comparable to ESM3. To understand how this is achieved, we systematically compare three different feature-fusion designs and fine-tuning recipes. Our results reveal that both the fusion method and the tuning strategy strongly affect final accuracy, indicating that the fine-tuning process is not trivial. We hope this work offers practical guidance for injecting structure into pretrained PLMs and motivates further research on better fusion mechanisms and fine-tuning protocols.

## Introduction

Proteins are vital macromolecules that perform a diverse array of cellular functions, from catalyzing biochemical reactions to maintaining structural integrity and regulating signaling pathways. These functions are determined by the protein’s three-dimensional structure, which in turn is encoded by its amino acid sequence [1, 2]. During natural evolution, mutations inevitably arise in protein sequences. While most are random, their long-term persistence is shaped by selective pressures that favor variants better adapted to their environments [3]. Changes at specific residues can significantly impact a protein’s folding stability, functional fitness, or biochemical activity [4–8]. While it sometimes leads to a complete loss of function or even toxic effects. Such mutational outcomes are central to both the emergence of new protein functions and the molecular basis of genetic diseases.

Deep mutational scanning (DMS) is an experimental technique that systematically measures the functional impact of a vast number of sequence variants for a given protein [9–11]. By introducing and testing millions of mutations, DMS generates high-resolution maps that link sequence changes to functional outcomes. These datasets have become invaluable for understanding sequence-function relationships, guiding protein engineering, and benchmarking computational prediction models. However, due to the high cost and limited throughput of DMS experiments, it is infeasible to apply them broadly across all proteins or mutation types [10]. To address this limitation, numerous computational methods have been proposed to predict mutational effects based on sequence features [12, 13], structural features [14–16], and evolutionary information [17–20]. Among these, multimodal protein language models (PLMs) have demonstrated strong generalization capabilities, leveraging large-scale unlabeled protein sequences to capture evolutionary and biochemical constraints without explicit supervision [21] (Fig. 1).

**Figure 1:**
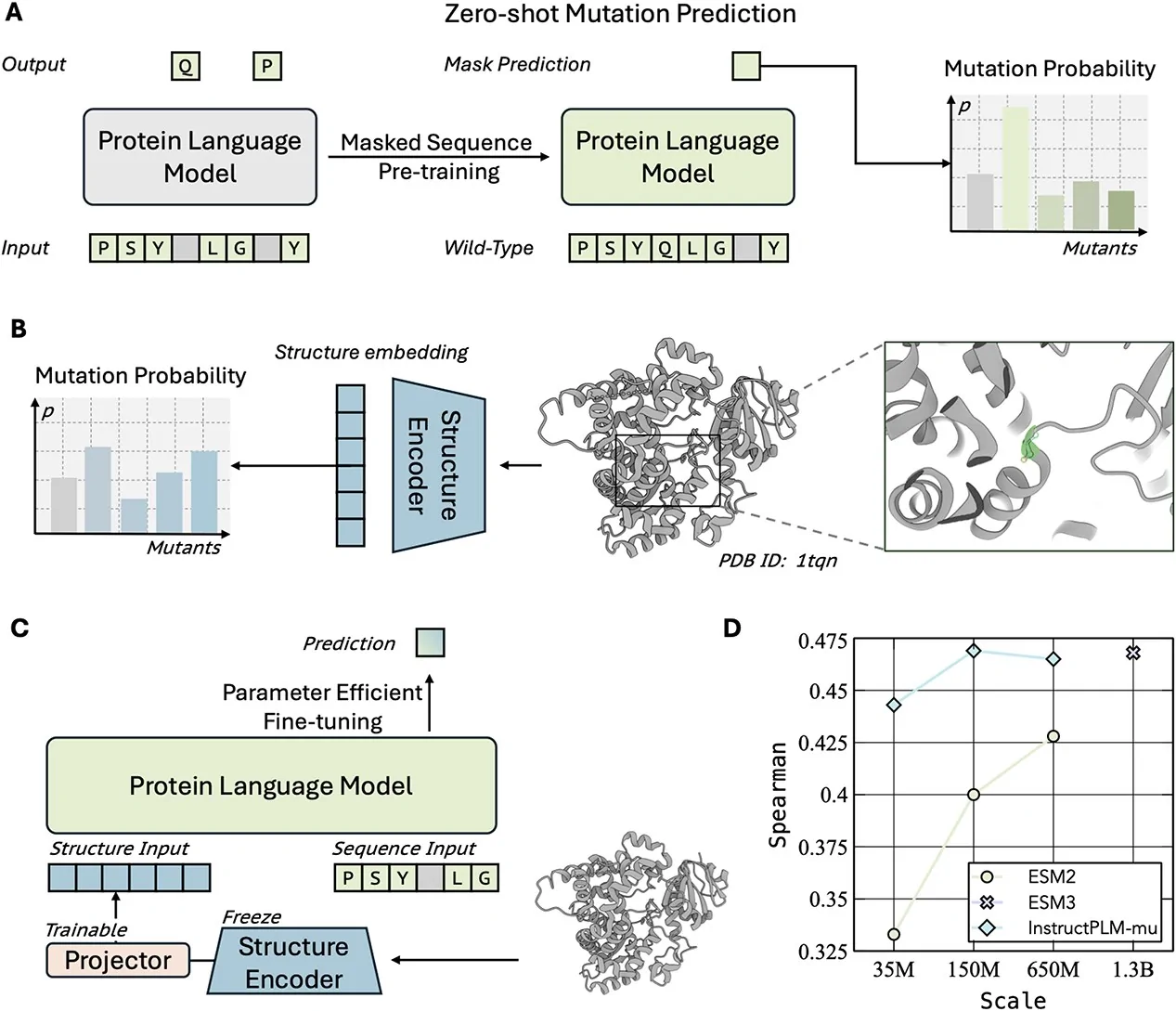
Overview of the InstructPLM-mu framework. (A) Zero-shot mutation prediction with PLMs. The PLM is pretrained on large-scale protein sequences and then used to predict mutation effects by computing the likelihood ratio between mutant and wild-type residues. (B) Residues in 3D contact, including those that are distant in sequence but close in space (long-range contacts), are essential for maintaining structural stability and functional integrity [29, 30]. Consequently, 3D structure-based representations provide a more sensitive characterization of the functional context of coding sites than sequence-based features alone. (C) InstructPLM-mu enables sequence-only PLMs to understand structural information through parameter-efficient fine-tuning. A lightweight projector maps structure embeddings into the PLM’s input space, and the model is trained with MLM while keeping most pretrained parameters frozen. (D) Performance comparison showing that InstructPLM-mu (150M) achieves comparable results to ESM3 (1.4B) with significantly lower computational cost.

However, training multimodal PLMs from scratch typically demands substantial computational resources and large-scale annotated datasets, making such approaches impractical for many researchers [22]. Inspired by recent advances in vision-language models, several studies have explored the use of pretrained PLMs as a backbone, and then fine-tuning them with additional modalities (e.g. structure or evolutionary data) [23–25]. These approaches have demonstrated promising results while remaining data- and resource-efficient. Nevertheless, how to effectively integrate new modalities into pretrained models remains an open research question, with design choices in fusion strategies playing a critical role in downstream performance.

In this paper, we aim to find an efficient way to fuse structure embeddings into PLMs, attempting to answer the question: *Can multimodal fine-tuning achieve comparable or even surpass the multimodal model trained from scratch?* Specifically, we propose a multimodal fine-tuning framework called InstructPLM-mu, and investigate three different strategies: Cross Attention, Channel-wise Concat, and Token-wise Concat. We apply our methods to the widely used PLM ESM2 [12] and two representative structure encoders, ProteinMPNN [26], a message-passing neural network that designs sequences from backbone structure, and ESM-IF [27] an inverse folding model built on the ESM protein language model, to evaluate the performance on the zero-shot protein mutation prediction task. Through extensive experiments, our results show that fine-tuned models can match the performance of advanced multimodal methods, including ESM3 [28], which is a newer, bigger, and multimodal successor of ESM2. An overview of this performance comparison is shown in Fig. 1. More importantly, our ablation shows that choosing the fine-tuning strategy is also critical; an overly aggressive training recipe may lead to knowledge forgetting of the pretrained PLMs. We will release code and checkpoints to facilitate reproducibility.

## Related works

### Protein mutation prediction

Protein mutation prediction is central to understanding protein function and guiding protein engineering. Classical approaches such as Rosetta [31] and ABACUS2 [32] rely on energy-based scoring, but are hindered by sampling limitations and biases in their underlying potentials. Deep learning has opened new directions: models like ESM-1v [17] predict mutation effects from large-scale sequence data, while structure-aware methods such as ProSST [33] and Pythia [16] further improve accuracy by incorporating structural information.

Benchmarking efforts such as ProteinGym [21] have underscored the power of pre-trained PLMs in protein modeling. ProteinGym evaluated over 250 DMS assays, revealing that PLMs effectively capture evolutionary constraints and generalize across diverse proteins. For instance, AIDO.Protein [20], a state-of-the-art PLM with a mixture-of-experts architecture, highlights the potential of PLMs to scale up protein modeling tasks with enhanced computational efficiency. Meanwhile, models like VenusREM [34] and S3F [15] leverage multimodal information—integrating structure, sequence, evolution, and surface features—to precisely model local conformational and energetic changes, which significantly boosts model performance.

### Multimodal alignment

Multimodal alignment aligns heterogeneous features across modalities to enhance cross-modal understanding and specific task performance [35]. This field has gained prominence with advances in large language and vision models [36]. Key challenges in multimodal learning include feature fusion and training paradigm, which are critical for model performance [37, 38].

#### Feature fusion

Previous studies on multimodal alignment largely focus on the way of projection between different modalities. For example, multi-layer perceptron (MLP) projection methods successfully bridge visual features to large language model (LLM) token spaces [39–41]. Query-based resampling optimizes computational efficiency through cross-attention compression of visual tokens [42]. Architectures with gated or sparse cross-attention layers for deeper multimodal integration [36, 43]. The main goal of these methods is to discuss how to deal with images with different resolutions and scales. However, recent work highlights that not only the manipulation of multimodal features but also the manner in which these features are fused inside the language model is crucial. DeepStack [44], for instance, demonstrates that multimodal performance can be enhanced by injecting vision features into multiple layers of the LLM. Similarly, a recent study systematically examines four different fusion strategies across a broad range of NLP tasks [45]. Despite these advances, multimodal fusion strategies remain underexplored in the context of PLMs. Notably, unlike vision–language models, protein structural features can be naturally aggregated at the residue level, which facilitates fine-grained integration of structural signals into sequence representations and opens up opportunities for designing more efficient and biologically informed fusion mechanisms.

#### Training paradigm

The end-to-end training paradigm jointly optimizes all parameters in a single phase, pursuing global optimization at the cost of high computational demand and potential suboptimal alignment due to limited intermediate refinement [37]. In contrast, multi-stage training separately fine-tunes modality-specific modules (e.g. image encoder) before full-model optimization, improving efficiency and final performance [39, 46, 47]. Unified pretraining integrates multimodal inputs within a single framework, typically employing masked or autoregressive objectives to achieve cross-modal fusion [48]. Additionally, strategies such as reinforcement learning have been proposed for supervision-efficient alignment in specific contexts [49, 50].

Inspired by advances in vision-language multimodal alignment, similar sequence-structure alignment strategies are now being adapted for protein modeling [14, 24, 28, 33]. Here, we compare multiple alignment mechanisms between pretrained sequence and structure modules, evaluating their performance on protein mutation prediction [21]. This task is central to protein engineering, as accurately predicting the functional effects of mutations (e.g. on stability, binding, and activity) requires high-quantity representation and deep integration of both sequence-structure relationships for precise functional inference.

## Methods

In this section, we compare three different multimodal fusion strategies and highlight their key differences. We denote the embedding of the amino acid sequence with length *L* (sequence length) as 


(1) \begin{align*}& \mathbf{X}^{(seq)} = [x_{1}, x_{2}, \dots, x_{L}], \quad x_{i} \in \mathbb{R}^{d_{s}},\end{align*}

where $d_{s}$ is the embedding dimension for sequence tokens after the embedding layer of PLMs. The corresponding protein structure encoder produces a structural embedding of the same sequence length 


(2) \begin{align*}& \mathbf{X}^{(str)} = [s_{1}, s_{2}, \dots, s_{L}], \quad s_{i} \in \mathbb{R}^{d_{t}},\end{align*}

where $d_{t}$ is the structural embedding dimension. Before the structure embeddings are fused to sequence embeddings, we use a learnable MLP $\mathbf{W}_{str} \in \mathbb{R}^{d_{t} \times d_{s}}$ to match the dimensions between modalities [39]: 


(3) \begin{align*}& \tilde{\mathbf{X}}^{(str)} = [\tilde{s}_{1}, \dots, \tilde{s}_{L}] = \mathrm{MLP}(\mathbf{X}^{(str)}), \quad \tilde{\mathbf{X}}^{(str)} \in \mathbb{R}^{L \times d_{s}}.\end{align*}

### Cross attention

In the Cross Attention strategy, we follow the implementation of LM-Design [23], inserting an additional Cross Attention sub-layer into the final Transformer block of the PLM (Fig. 2 left). Let *T* denote the total number of Transformer blocks in the PLM, and let $\mathbf{H}^{(t)} \in \mathbb{R}^{L \times d_{s}}$ be the sequence representation output from the *t*th block. In particular, $\mathbf{H}^{(T-1)}$ is the output from the penultimate block (i.e. before the final block). The final block is modified to include an additional Cross Attention branch: 


(4) \begin{align*} & \mathbf{H}^{(seq)}_{\mathrm{self}} = \mathrm{SelfAttn}(\mathbf{H}^{(T-1)})\end{align*}

(5) \begin{align*}& \mathbf{H}^{(seq)}_{\mathrm{cross}} = \mathrm{CrossAttn}\!\left( \begin{aligned} &Q = \mathbf{H}^{(seq)}_{\mathrm{self}},\\ &K = \tilde{\mathbf{X}}^{(str)},\\ &V = \tilde{\mathbf{X}}^{(str)} \end{aligned} \right)\end{align*}

(6) \begin{align*}& \mathbf{H}^{(T)} = \mathbf{H}^{(seq)}_{\mathrm{self}} + \mathbf{H}^{(seq)}_{\mathrm{cross}}\end{align*}

**Figure 2:**
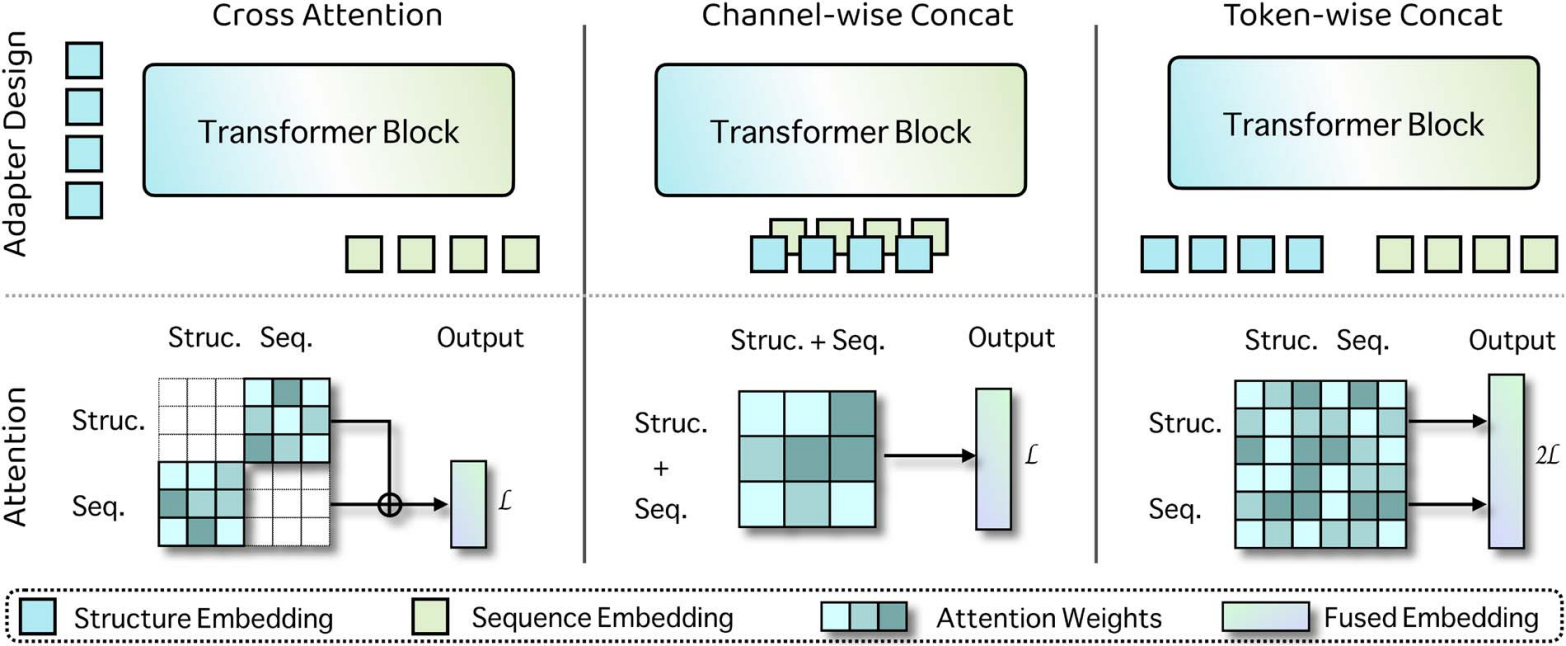
Comparison of three multimodal fusion strategies: Cross Attention (left), which inserts a cross-attention sub-layer where sequence tokens attend to structure tokens as key and value; Channel-wise Concat (middle), which performs element-wise addition of projected structure embeddings to sequence embeddings; and Token-wise Concat (right), which treats structure embeddings as additional input tokens, enabling bidirectional attention between modalities at the cost of doubling the sequence length (L *→ * 2L).

The fused representation $\mathbf{H}^{(T)}$ is then passed to the PLM’s output layer for prediction.

The Cross Attention design has several limitations. First, since the structure embeddings $\tilde{\mathbf{H}}^{(str)}$ serve only as the key and value in the Cross Attention operation, there is *no self-attention mechanism among structural tokens themselves*. This prevents the structure modality from refining its internal representation or capturing long-range dependencies purely within the structural space during fusion. Second, this one-way attention flow (sequence *→ * structure) does not allow reciprocal updates from structure to sequence across layers; consequently, the structure features will not merge information from sequences.

### Channel-wise concat

In the Channel-wise Concat strategy, structural features are directly merged with sequence features at the embedding level; this strategy is adopted by ESM 3 [28]. (Fig. 2 middle.) Specifically, the projected structure embeddings are added element-wise to the sequence embeddings: 


(7) \begin{align*}& \mathbf{Z} = \mathbf{X}^{(seq)} + \tilde{\mathbf{X}}^{(str)}, \quad \mathbf{Z} \in \mathbb{R}^{L \times d_{s}}.\end{align*}

The fused representation $\mathbf{Z}$ is fed into the PLM in place of the original sequence embeddings.

Compared to Cross Attention, Channel-wise concatenation enables attention on both structure and sequences: when the PLM performs self-attention over $\mathbf{Z}$, information from the structure and sequence modalities can flow jointly. However, the tight coupling also means there is no mechanism for *selective* information flow, structural features cannot be dynamically weighted or ignored depending on context. In other words, the model treats the sum of sequence and structure features as a single representation, which may lead to suboptimal integration when one modality contains noisy or task-irrelevant information.

### Token-wise concat

In the Token-wise Concat strategy, structural embeddings are treated as additional input tokens, enabling the PLM to process sequence and structure jointly through its self-attention mechanism. (Fig. 2 right.) Given the projected structure embedding $\tilde{\mathbf{X}}^{(str)}$, we concatenate the structural tokens and the sequence tokens along the sequence dimension: 


(8) \begin{align*}& \mathbf{Z} = [\tilde{s}_{1}, \dots, \tilde{s}_{L}, x_{1}, \dots, x_{L}] \in \mathbb{R}^{2L \times d_{s}}.\end{align*}

To ensure alignment between modalities, we assign the same position index to $\tilde{s}_{i}$ and $x_{i}$, so that the *i*th structural token corresponds to the *i*th amino acid in the sequence.

We refer to this design as InstructPLM-mu, highlighting its ability to inject *instruction-like* structural tokens into the PLM. Unlike channel-wise fusion, which enforces a static combination of modalities, InstructPLM-mu enables dynamic, bidirectional information flow between sequence and structure tokens through self-attention. This design allows the model to flexibly attend to or ignore structural cues depending on context, thereby offering richer cross-modal interactions. However, doubling the sequence length increases computational cost and memory usage, particularly for large sequence length *L*.

### Training target

For all three fusion strategies, we adopt a masked language modeling (MLM) objective applied to the protein sequence. Given a wild-type amino acid sequence $\mathbf{X}^{(seq)}$, we randomly select a subset of positions $\mathcal{M} \subset \{1, \dots , L\}$ to mask. The corresponding residues are replaced with a special [MASK] token in the sequence branch (align with the original PLM), while the structural tokens remain unchanged. The model is trained to recover the original amino acid at each masked position: 


(9) \begin{align*}& \mathcal{L}_{\mathrm{MLM}} = - \sum_{i \in \mathcal{M}} \log p_\theta \big(x_{i} \,\big|\, \mathbf{X}_{\setminus \mathcal{M}}^{(seq)}, \mathbf{X}^{(str)} \big)\end{align*}

where $p_\theta $ denotes the output distribution of the PLM with fused modalities. This setup forces the model to leverage structural context to reconstruct masked residues, encouraging it to learn complementary relationships between sequence and structure.

### Zero-shot protein mutation prediction

We use masked-marginals [17] to calculate the mutation score. Let $\mathbf{X}^{(seq,wt)}$ be the wild-type sequence and $\mathbf{X}^{(seq,mut)}$ a mutant; let $\mathcal{M}$ be the set of mutated positions. For each $i\in \mathcal{M}$ we form a masked input by replacing the residue at *i* with $\mathtt{[MASK]}$ in the sequence branch while keeping structural tokens unchanged. The per-site score is 


(10) \begin{align*}& \begin{aligned} s_{i} ={}& \log p_\theta\!\big(x_{i}^{(mut)} \mid \mathbf{X}_{\setminus i}^{(seq,mut)}, \mathbf{X}^{(str)}\big) \\ &- \log p_\theta\!\big(x_{i}^{(wt)} \mid \mathbf{X}_{\setminus i}^{(seq,mut)}, \mathbf{X}^{(str)}\big). \end{aligned}\end{align*}

The final mutant score is the sum over mutated sites: 


(11) \begin{align*}& S(\mathbf{X}^{(seq,mut)}) \;=\; \sum_{i\in\mathcal{M}} s_{i}.\end{align*}

Higher *S* indicates the model favors the mutant residues over the wild type under the given structural context.

## Experiments

### Implementation details

We train InstructPLM-mu using the CATH 4.3 dataset [51] and tested on the ProteinGYM benchmark [21]. We randomly split the CATH 4.3 dataset into train and validation with a ratio of 9:1, and perform checkpoint selection using the validation loss. To accelerate the fine-tuning process, we crop the training sequence to a maximum of 512 tokens, as the token-wise concat strategy increases training cost. Notably, as our downstream task does not involve testing on CATH 4.3, we thus do not adopt a test split of CATH 4.3. We evidence the model’s ability by examining the prediction scores of the model for mutation outcomes (11) and experimental scores. The original ProteinGYM benchmark comprises 217 assays. Because most baseline models can process protein sequences only up to 1024 residues, we excluded proteins longer than 1000 residues, resulting in a final set of 201 assays. Details of the datasets are provided in Table 1. All experiments are done with 4 Nvidia A100 GPUs; additional evaluation metrics, training hyperparameters, and computational efficiency details are provided in Supplementary Tables S1 and S2.

**Table 1:** Summary of datasets used in InstructPLM-mu.

	**CATH 4.3**		**ProteinGYM**				
Dataset	Train	Validation	Acitivity	Binding	Expression	Fitness	Stability
Number	22727	2525	39	12	16	69	66

The training and validation sets are derived from CATH 4.3, while evaluation is performed on the ProteinGYM benchmarks, covering activity, binding, expression, fitness, and stability. The table lists the number of sequences or mutational assays in each split.

### Main results

#### Token-wise concatenation emerges as the superior fusion strategy

In order to understand how fusion strategies influence the performance of multimodal fine-tuning, we first investigate the performance across three different structure fusion strategies. Table 2 summarizes the results of zero-shot mutation effect prediction.

**Table 2:** Comparison of multimodal fine-tuning strategies against single-modal PLMs on zero-shot mutation effect prediction.

**Method**	**Average**	**Activity**	**Binding**	**Expression**	**Fitness**	**Stability**
ESM2 (35M)	0.333	0.325	0.32	0.357	0.224	0.437
ESM2 (150M)	0.4	0.405	0.358	0.422	0.308	0.507
ESM2 (650M)	0.428	0.44	0.369	0.44	0.372	0.52
ESM2 (3B)	0.421	0.434	0.351	0.429	0.382	0.507
Channel-wise concat	0.435	0.438	0.394	0.443	0.378	0.524
Cross attention	0.44	0.453	0.329	0.428	**0.394**	0.597
Token-wise concat	**0.469**	**0.462**	**0.414**	**0.466**	0.389	**0.614**
Relative Gain	9.5%	5.0%	12.2%	5.9%	3.1%	18.1%

The reported values are Spearman correlation coefficients; higher values indicate better predictive performance, best and second best results are shown in **bold** and underlines respectively.

Among three fusion strategies, Token-wise Concat obtains the highest average score (0.469). It also achieves the best performance in four out of five functional categories, showing that this design not only improves the overall average but also delivers stable gains across different evaluation aspects. This suggests that treating structural embeddings as extra tokens allows the model to use them more flexibly, depending on the context. Notably, although Channel-wise concat and Cross Attention reach similar average scores (0.435 versus 0.440), their strengths appear in different places. For example, cross attention performs a strong correlation on *activity* and even achieves a higher score in *fitness* of Token-wise Concat, while Channel-wise Concat gives stronger results on *binding* and *expression*. This divergence suggests that the way structural features are integrated can have very different effects depending on the functional property being predicted. Overall, these results highlight the importance of fusion design: while simply incorporating structure boosts performance, enabling flexible, token-level integration of modalities yields the most consistent and robust improvements across diverse protein mutation prediction tasks. Statistical significance analysis for the main comparison is reported in Supplementary Table S3, and per-assay performance across individual DMS datasets is shown in Supplementary Figs S1–S3.

#### InstructPLM-mu consistently outperforms single-modal sequence models

When comparing multimodal approaches against the sequence-only baselines, we observe clear and consistent improvements. For average performance, all three fusion strategies surpass ESM2 models of different scales, showing that incorporating structure is more effective than simply enlarging the backbone. Specifically, our methods achieved an 18.1% improvement in the stability function (from 0.52 to 0.614). In fact, the best and second-best results in every functional category are achieved by fine-tuned multimodal models, underscoring the strong advantage of leveraging structure in this setting. These results demonstrate that structural context is a key driver of performance in mutation effect prediction, and incorporating it through fine-tuning offers a more reliable path forward than scaling sequence-only PLMs.

#### Multimodal fine-tuning achieves competitive results with only a fraction of the resources

To understand whether InstructPLM-mu can catch multimodal methods that are trained from scratch, we make a comparison of the current SOTA structure-sequence methods, including SaProt [14], ESM3 [28], S3F [15], and ProSST [33]. As Table 3 shows, while methods trained from scratch obtain strong performance, they typically require an extensive training cost. For example, ProSST is trained on 8*A100 GPUs for a month [33]. In contrast, our fine-tuned models build on existing models and require efficient computational capacity, not only to close the gap but also to surpass larger, scratch-trained baselines. Notably, Token-wise concat (0.469) achieves comparable performance to ESM3 (0.468), despite ESM3 being a 1.4B parameter model trained with far greater resources.

**Table 3:** Comparison of InstructPLM-mu with SOTA structure-sequence methods.

**Method**	**Average**	**Activity**	**Binding**	**Expression**	**Fitness**	**Stability**
MULAN [52]	0.323	0.298	0.344	0.376	0.232	0.366
MIF [53]	0.382	0.337	0.320	0.420	0.310	0.521
MIF-ST [53]	0.400	0.409	0.306	0.428	0.375	0.483
Channel-wise Concat	0.435	0.438	0.394	0.443	0.378	0.524
ESM-IF [27]	0.440	0.418	0.388	0.437	0.333	0.623
Cross Attention [23]	0.440	0.453	0.329	0.428	0.394	0.597
ProtSSN [54]	0.453	0.475	0.373	0.452	0.399	0.566
S2F [15]	0.460	0.474	0.394	0.462	0.403	0.566
SaProt [14]	0.462	0.477	0.380	0.486	0.377	0.591
ESM3 [28]	0.468	0.449	0.395	0.466	0.391	0.640
Token-wise Concat	0.469	0.462	0.414	0.466	0.389	0.614
S3F [15]	0.473	**0.483**	0.403	0.474	0.413	0.592
ProSST [33]	**0.506**	0.479	**0.435**	**0.521**	**0.441**	**0.651**

Results of InstructPLM-mu are shown in gray. The reported values are Spearman correlation coefficients; higher values indicate better predictive performance, best and second best results are shown in **bold** and underlines respectively.

S2F and S3F also leverage multimodal information for mutation prediction but follow a different technical approach: they extract frozen ESM2 embeddings and train separate encoders to combine structural and sequence features. In contrast, InstructPLM-mu directly fine-tunes ESM2 to understand structural information. Despite this architectural difference, our method achieves better performance (0.469 versus 0.460) with lower training cost (4 h versus 9 h for S2F [15]). S3F achieves higher performance by incorporating additional surface information, which aligns with our finding that multimodal integration improves mutation prediction. Among fine-tuned models using only sequence and structure information (ProtSSN, S2F), our token-wise concatenation approach achieves the best performance. ProSST, which outperforms all other methods, is trained from scratch with substantially more computational resources.

Another interesting observation is that our methods consistently boost the standalone performance of the original structure encoder ESM-IF on 4 out of 5 different functions, showing that structural and sequence features reinforce each other rather than being passively combined. In summary, these results demonstrate that fine-tuning with modality fusion offers a resource-efficient yet highly effective alternative to training multimodal PLMs from scratch. Additional generalization tests after removing potential training-test overlap are summarized in Supplementary Table S4.

### Fine-tuning strategies

To teach the pretrained PLMs to understand structures, a new adapter has been introduced to connect two different modalities (Equation (3)), and the three corresponding training recipes are summarized schematically in Fig. 3. This raises the question of how to train the added parameters: Do different training recipes influence the final performance? To answer it, we evaluate three fine-tuning strategies: Full Fine-tune, low-rank adaptation (LoRA) [55] + Adapters, and Adapter-only. LoRA is a parameter-efficient fine-tuning method that injects trainable low-rank matrices into transformer layers, enabling efficient adaptation while preserving pretrained knowledge. These strategies differ in the fraction of tunable parameters and thus reflect different degrees of intervention on the pretrained model. Specifically, Adapter-only updates only the adapter parameters ( 1% of total parameters), directly controlling how structural embeddings are injected without modifying the backbone. LoRA + Adapters additionally applies low-rank updates to selected PLM layers ( 5%–10% of total parameters), offering a middle ground between efficiency and capacity. Detailed trainable parameters for each configuration are provided in Supplementary Table S2. Full Fine-tune updates all parameters of the PLM and adapters, yielding maximum flexibility at the highest computational cost.

**Figure 3:**
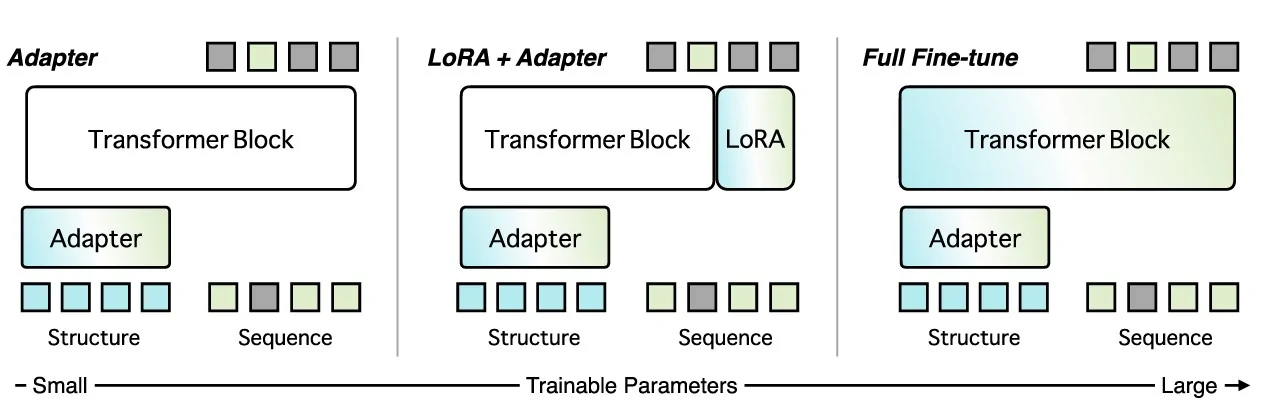
Schematic of the three fine-tuning strategies: Adapter-only (left), where the backbone is frozen and only the adapters projecting structural features into the PLM are learned; LoRA + Adapter (middle), where adapters inject structural embeddings while low-rank (LoRA) updates are applied to selected transformer weights; and Full Fine-tune (right), where all transformer blocks and adapter modules are updated.

As Table 4 shows, the first important message is that more tunable parameters *do not* correspond to better performance. Concretely, in the Token-wise Concat method, the LoRA + Adapter strategy performs best across all three scales of the pre-trained model, followed by Full Fine-tune and Adapter-only strategy. In the Channel-wise Concat method, however, excessive adjustable parameters can even severely impair performance, suggesting that over-tuning the model can destroy previously learned knowledge during pre-training. Additional LoRA-rank ablations for the unstable 650M Channel-wise Concat setting are reported in Supplementary Table S5. Secondly, the optimal fine-tune strategy is not generalizable and can be affected by the feature fusion method and even the model size. For example, the LoRA + Adapter strategy performs best in the Token-wise Concat method, while in the Channel-wise Concat method, the LoRA + Adapter strategy (0.296) is worse than the Full Fine-tune (0.305) and the Adapter-only (0.311) methods in the 35M backbone. In terms of the 150M backbone, the LoRA + Adapter strategy obtained the best performance (0.395) with respect to the Full Fine-tune (0.378) and Adapter-only (0.385) strategies, reflecting the high variance of the same fine-tuning strategy on different fusion methods and scales of the backbone model.

**Table 4:** Comparison of fine-tuning strategies across model scales (35M, 150M, 650M) and fusion methods (Token-wise versus Channel-wise).

**Fine-tune strategy (tunable parameters)**	**Token-wise Concat**			**Channel-wise Concat**		
	35M	150M	650M	35M	150M	650M
ESM2	0.333	0.4	0.425	0.333	0.4	0.425
Full Fine-tune (100%)	0.435	0.456	0.463	0.305	0.378	0.029
LoRA + Adapter (5%–10%)	0.443	0.469	0.465	0.296	0.395	0.005
Adapter-only (*∼ *1%)	0.358	0.407	0.45	0.311	0.385	0.435

Values are Spearman correlation coefficients averaged across all 201 assays in the ProteinGYM benchmark.

Third, clear scaling behavior, i.e. consistent performance improvements as the backbone grows, appears primarily under the Adapter-only setting. While prior work has reported benefits from increasing pre-trained model scale for multimodal fine-tuning [56, 57], our experiments show that this monotonic scaling is mainly observed when only the adapters are tuned. The results of the Adapter-only strategy show a clear increase path as the model scales, both on Token-wise Concat and Channel-wise Concat. For LoRA+Adapter and Full Fine-tune, gains are often present for smaller backbones but can stagnate or become unstable on larger ones, suggesting potential overfitting or interference with pretrained weights.

### Ablation on main components

To understand how component design influences the performance, we conducted ablations on adapter layers and structure encoders. Table 5 reports controlled ablations on two axes: the depth of the MLP used to project structural features, and the choice/combination of structure encoders. All experiments are performed on the 35M backbone, and trained using the LoRA + Adapter strategy.

**Table 5:** Ablation results on the 35M backbone with LoRA + Adapter tuning.

		**Average**	**Activity**	**Binding**	**Expression**	**Fitness**	**Stability**
Layers	2 *× * MLP	0.443	0.413	0.387	0.449	0.35	0.614
	3 *× * MLP	0.443	0.412	0.384	0.45	0.351	0.617
	4 *× * MLP	0.441	0.411	0.384	0.445	0.349	0.614
Encoder	ProteinMPNN	0.42	0.404	0.352	0.429	0.327	0.589
	ESM-IF	0.443	0.412	0.384	0.45	0.351	0.617
	ProteinMPNN + ESM-IF	0.437	0.408	0.379	0.445	0.343	0.609

The top block varies the depth of the projection MLP used to map structural embeddings. The bottom block compares structure encoders. For the ProteinMPNN + ESM-IF combination, we concatenate the embeddings from both encoders along the feature dimension before projecting to the sequence embedding space.

For the MLP depth, results are very close across 2, 3, and 4 layers. There is no clear improvement as we add depth: 2*× *MLP and 3*× *MLP give essentially the same average performance, while 4*× *MLP shows a tiny drop. Task-wise differences are also minor (3*× *MLP marginally helps expression and stability), but the overall gains are negligible compared with the extra parameters and computational cost. Practically, a shallow MLP is sufficient and more efficient; based on this, we use 3 layers as our default setting.

For the structure encoder, ESM-IF is the strongest single encoder in our setup, improving average performance and several tasks relative to ProteinMPNN.

Interestingly, concatenating both encoders does not produce additive gains; the combined setup performs slightly worse than ESM-IF alone on most metrics. This suggests that ProteinMPNN and ESM-IF may capture overlapping structural information, and simple concatenation without gating or attention mechanisms can introduce noise. This scenario is also found in computer vision, where multi-encoder fusion has been extensively studied, with different approaches for integrating features from multiple encoders. For example, [58] and [59] concatenate embeddings from different encoders as additional tokens, while [60] and [61] explore channel-wise concatenation with gating mechanisms. While the primary focus of this work is exploring how to integrate structural features into PLMs rather than combining multiple encoders, we believe that developing more sophisticated multi-encoder fusion strategies represents a promising direction for future work.

In summary, the ablations indicate that using a stronger, better-aligned structure encoder is a promising direction; we therefore leave more systematic exploration of encoder combinations and smarter fusion mechanisms (e.g. per-encoder gating or attention-based fusion) to future work.

## Discussion

In this paper, we show that multimodal fine-tuning of pretrained PLMs is a practical, effective way to bring structural information into PLMs. Specifically, we introduce InstructPLM-mu and evaluate three fusion designs, including Cross Attention, Channel-wise Concat, and Token-wise Concat. Our experiments demonstrate that multimodal fine-tuning consistently improves zero-shot mutation prediction over sequence-only baselines, and is competitive with several stronger multimodal methods trained from scratch. Notably, we find that the choice of fusion is critical: Token-wise Concat delivers the most robust gains across multiple backbone scales and different downstream functional categories. Furthermore, extensive ablations evidenced that the fine-tuning recipe is also crucial: parameter-efficient schemes (e.g. LoRA + adapters) often provide the best trade-off between performance and cost, whereas overly aggressive updates (full fine-tuning) can harm larger backbones and lead to catastrophic forgetting.

From a biological perspective, our findings have important implications for protein engineering. The superior performance of Token-wise Concat suggests that treating structural information as “instruction-like” tokens allows the PLM to selectively attend to structural cues depending on context. This mirrors how structural biologists use structural information—not as a rigid constraint, but as contextual guidance that informs the interpretation of sequence variation. The residue-level alignment between sequence and structure tokens enables the model to capture local structural constraints that are most relevant for predicting mutation effects on protein stability and function.

Despite these positive results, several limitations point to clear directions for future work. First, stronger or more diverse structural encoders and better encoder-level fusion (e.g. per-encoder gating or attention) may unlock further gains beyond what simple concatenation provides; we leave systematic exploration of such fusion mechanisms to future work. Second, more fine-grained tuning protocols can be investigated. Such as staged training schedules, layer-wise unfreezing, or hybrid update schemes, can be performed to address the instability and catastrophic forgetting we observe when adapting very large backbones. Third, we test our framework on the ESM2 backbone with structure inputs. Leveraging stronger backbones (e.g. ESM3, SaProt, etc.) and extra modalities (e.g. multiple sequence alignments (MSAs)) is also a promising direction.

We consider these directions important next steps to broaden and solidify the practical utility of multimodal fine-tuning for protein modeling.

**Key points**

- Large multimodal protein models (e.g. ESM3) achieve strong mutation-effect prediction performance but require substantial computational resources for pretraining, limiting their accessibility.
- We demonstrate that lightweight multimodal fine-tuning of a sequence-only PLM (ESM2), by injecting residue-level structural embeddings, can achieve performance comparable to or exceeding that of fully pretrained multimodal models.
- Our framework, InstructPLM-mu, systematically evaluates multiple fusion strategies and fine-tuning recipes, providing practical guidance for effective multimodal adaptation.
- This work highlights multimodal fine-tuning can serve as a strong and effcient alternative to multimodal pretraining for protein mutation prediction tasks.

## Supplementary Material

InstructPLM_mu_supp_bbag400

## Data Availability

All data and code used in this study are publicly available to ensure reproducibility. The training dataset CATH 4.3 [51] and the evaluation benchmark ProteinGYM [21] are publicly accessible. For the baseline results reported in Table 3, We used the official ProteinGym release of “Zero-Shot DMS Assay Scores,” available at https://proteingym.org/download. The implementation of InstructPLM-mu and related resources, including model weights, are available at https://github.com/ssyypp1/InstructPLM-mu and https://huggingface.co/ssyypp111/ESM2-150M-TokenWise.
