# Supplementary material for: InstructPLM-mu: protein fitness prediction from structure instructions
Source: Brief Bioinform. 2026 Oct 9;27(5):bbag400. doi: 10.1093/bib/bbag400 (PMC13652651; doi:10.1093/bib/bbag400)
Supplement: Supplementary file 1 [file instructplm_mu_supp_bbag400.pdf]

Briefings in Bioinformatics, 2026, pp. 1–2
DOI added during production
Published: Date added during production
Supplementary Material

Supplementary Material for InstructPLM-mu: Protein Fitness Prediction from Structure Instructions

Junde Xu,[1,†] Yapin Shi,[3,†] Lijun Lang,[1] Taoyong Cui,[1] Zhiming Zhang,[2,4] Guangyong Chen,[2] Jiezhong Qiu[2,*] and Pheng-Ann Heng[1]

[1]The Chinese University of Hong Kong, Hong Kong SAR, China
[2]Hangzhou Institute of Medicine, Chinese Academy of Sciences, Hangzhou, Zhejiang, China
[3]Hangzhou Institute for Advanced Study, University of Chinese Academy of Sciences, Hangzhou, Zhejiang, China
[4]Tianjin University, Tianjin, China

[*]Corresponding author. jiezhongqiu@outlook.com [†]These authors contributed equally to this work.

Abstract

Training Details

Backbone model. We adopt the publicly released ESM2 protein language models as our backbone. Three different parameter scales are explored: 35M[1],150M[2], and 650M.[3] All checkpoints are initialized from the official HuggingFace releases without additional pre-training.

Structure encoder. We adopt the publicly released ProteinMPNN[4] and ESM-IF[5] (ESM-Inverse Folding) checkpoints as our structure encoders. Specifically, we concat 4 vanilla models of ProteinMPNN `v_48_002.pt, v_48_010.pt, v_48_020.pt, v_48_030.pt`. Input 3D coordinates are preprocessed following the official ProteinMPNN pipeline (atom type filtering and coordinate centering).

Multi-model Projector. To integrate sequence and structural representations, we employ a lightweight multi-modal projector implemented as a multi-layer perceptron (MLP) with GELU [1] activation. The projector maps the concatenated embeddings from the protein language backbone and the structure encoder into a unified latent space with a dimension of the backbone model. Layer normalization is applied after each hidden layer.

Low-Rank Adapter. For efficient fine-tuning of the backbone model, we insert Low-Rank Adaptation (LoRA) modules into every linear layer of the transformer blocks. The LoRA rank is set to 32 and the scaling factor (α) to 256. The adapters are trained jointly with the projector while all original backbone weights remain frozen.

1 https://huggingface.co/facebook/esm2_t12_35M_UR50D
2 https://huggingface.co/facebook/esm2_t30_150M_UR50D
3 https://huggingface.co/facebook/esm2_t33_650M_UR50D
4 https://github.com/dauparas/ProteinMPNN
5 https://huggingface.co/facebook/esm_if1_gvp4_t16_142M_UR50

Table S1 Summary of training hyperparameters for InstructPLM-mu.

Hyperparameter	Value
Learning rate	1e-4
Batch size	256
Number of training epochs	20
Optimizer	Adam
Warm-up steps	100
Weight decay	1e-1

Table S2 Computational efficiency analysis of InstructPLM-mu training.

Trainable/Total Params.	Batch Size	Epoches	Training Time
4M / 35M	256	20	∼30 min
12M / 150M	256	20	∼1 hour
27M / 650M	256	20	∼4 hours

Other Hyperparameters. Key hyperparameters used in training are summarized in Table S1. Detailed computational efficiency analysis is provided in Table S2.

Evaluation and More Results

Metrics. Model performance is evaluated using the Spearman rank correlation coefficient (Spearman's ρ) between the predicted mutation effects and the experimentally measured ground-truth scores. Spearman's ρ measures the monotonic relationship between two variables and is insensitive to the absolute scale of the predictions, making it well-suited for assessing whether the model correctly ranks protein variants by functional effect rather than merely matching their exact values.

© The Author(s) 2026. Copyright and licence statements to be updated by the publisher during production.

Statistical Significance Testing. To assess the statistical significance of performance differences, we follow the methodology used in ProteinGYM benchmarks to compute the standard error of the difference in Spearman correlation relative to the baseline methods. As Table S3 shows, the small standard errors relative to the differences indicate that our performance comparisons are statistically reliable.

Table S3 Standard error of Spearman correlation differences for Token-wise Concat (0.469).

Comparison	S2F	S3F	ProSST	ESM3
Δ Spearman	+0.009	-0.004	-0.037	+0.001
Std. Error of Diff.	0.003	0.002	0.003	0.001

Generalization Test. To further validate the generalization capability of our approach, we conducted additional experiments after removing training sequences with different levels of identity to any test sequence in ProteinGYM. Specifically, We use FoldSeek [2] to filter out proteins with structure similarity > 0.4, 0.6, and 0.8, and MMseqs2 [3] for sequence similarity filtering at the same thresholds. , respectively, and retrain our best config, 150M backbone with ESM-IF encoder. As shown in Table S4, the performance remains consistent after deduplication, with only minor variations. This demonstrates that our results are not affected by potential data leakage and that the model generalizes well to unseen proteins.

Table S4 Performance comparison with and without training-test overlap. Results show Spearman correlation on ProteinGYM after removing training proteins with different levels of identity to test sequences.

Identity	0.4	0.6	0.8	1.0
# Train Seq.	24609	25069	25106	25252
Spearman	0.466	0.471	0.468	0.469

To further investigate the instability of Channel-wise Concat with larger backbones, we conducted an ablation study varying the LoRA rank. As shown in Table S5, reducing the LoRA rank from 32 to 4 for the 650M backbone with Channel-wise Concat consistently yields marginal performance (Spearman ρ ranging from -0.032 to 0.074). However, the persistent failure across this range of ranks indicates that the collapse is a reproducible bottleneck for Channel-wise Concat at this model scale. In contrast, Token-wise Contact strategy maintain robust performance at the same scale, reflecting their superior compatibility and their capacity to preserve pre-trained knowledge during fine-tuning.

Results per assay. For each assay, we report the Spearman correlation across all tested variants. Figures S1, S2, and S3 show the performance of the baseline models (ESM2 (650M) and ESM3) as well as our fine-tuned methods. For clarity, we highlight the results of the Token-wise Concat model with the ESM-IF encoder and trained with the LoRA + adapter strategy. The figures indicate that the improvements are most pronounced on proteins for which the baseline model (ESM2) previously performed poorly (Figure S1), while still maintaining relatively high correlation on assays that were easier for the baseline (Figure S3).

Table S5 Effect of LoRA rank on Channel-wise Concat (650M backbone). Lower ranks indicating the lower number of trainable parameters

LoRA Rank	4	8	16	32
Spearman	0.074	0.072	-0.032	0.005

References

1. Dan Hendrycks and Kevin Gimpel. Gaussian error linear units (gelus). *arXiv preprint arXiv:1606.08415*, 2016.
2. Michel Van Kempen, Stephanie S Kim, Charlotte Tumescheit, Milot Mirdita, Jeongjae Lee, Cameron LM Gilchrist, Johannes Söding, and Martin Steinegger. Fast and accurate protein structure search with foldseek. *Nature biotechnology*, 42(2):243–246, 2024.
3. Martin Steinegger and Johannes Söding. Mmseqs2 enables sensitive protein sequence searching for the analysis of massive data sets. *Nature biotechnology*, 35(11):1026–1028, 2017.

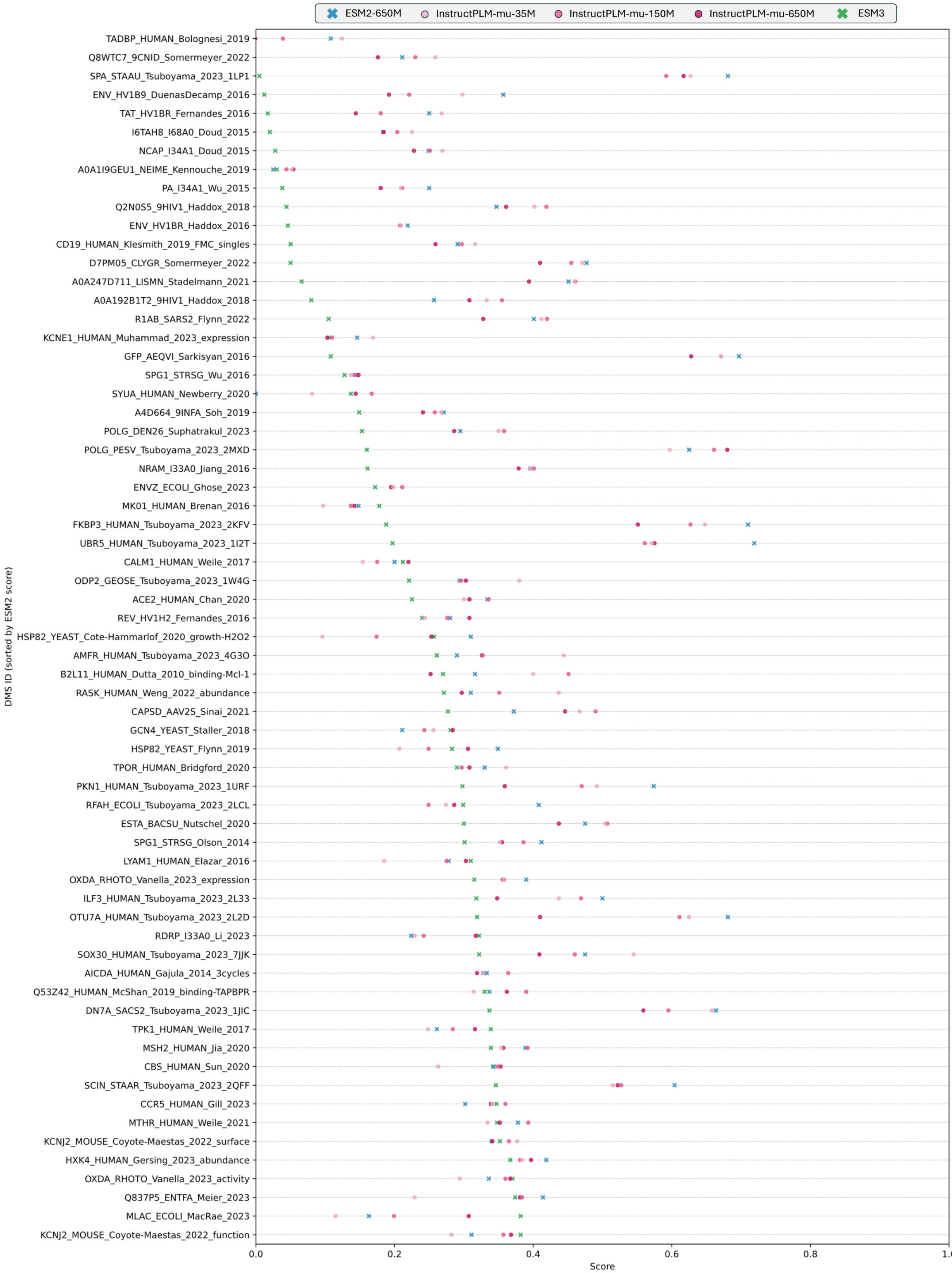

Figure S1 Spearman correlations on individual DMS datasets, sorted by ESM3 performance. Baselines use cross markers; InstructPLM-mu are shown as colored circles.

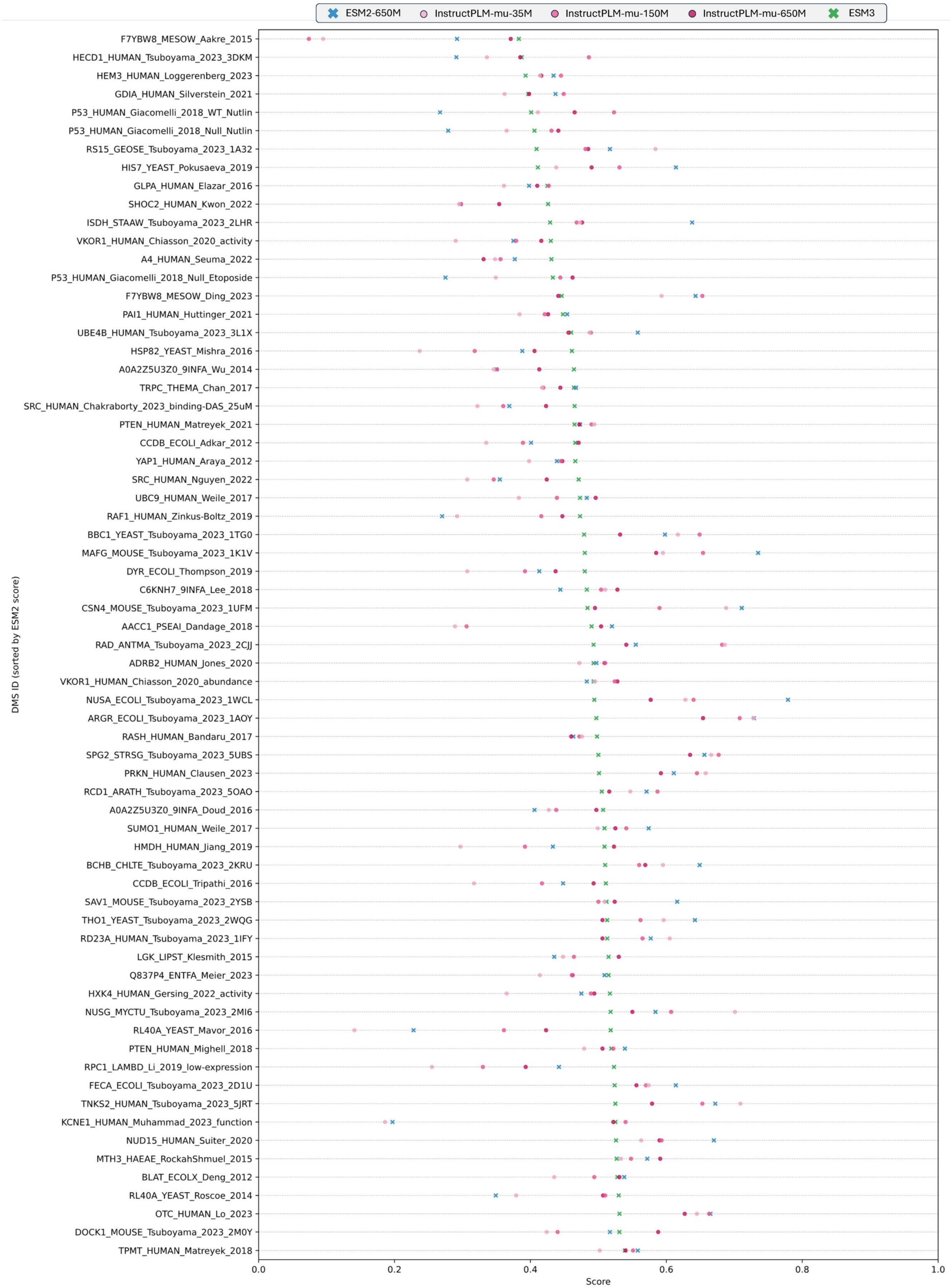

Figure S2 Spearman correlations on individual DMS datasets, sorted by ESM3 performance. Baselines use cross markers; InstructPLM-mu are shown as colored circles.

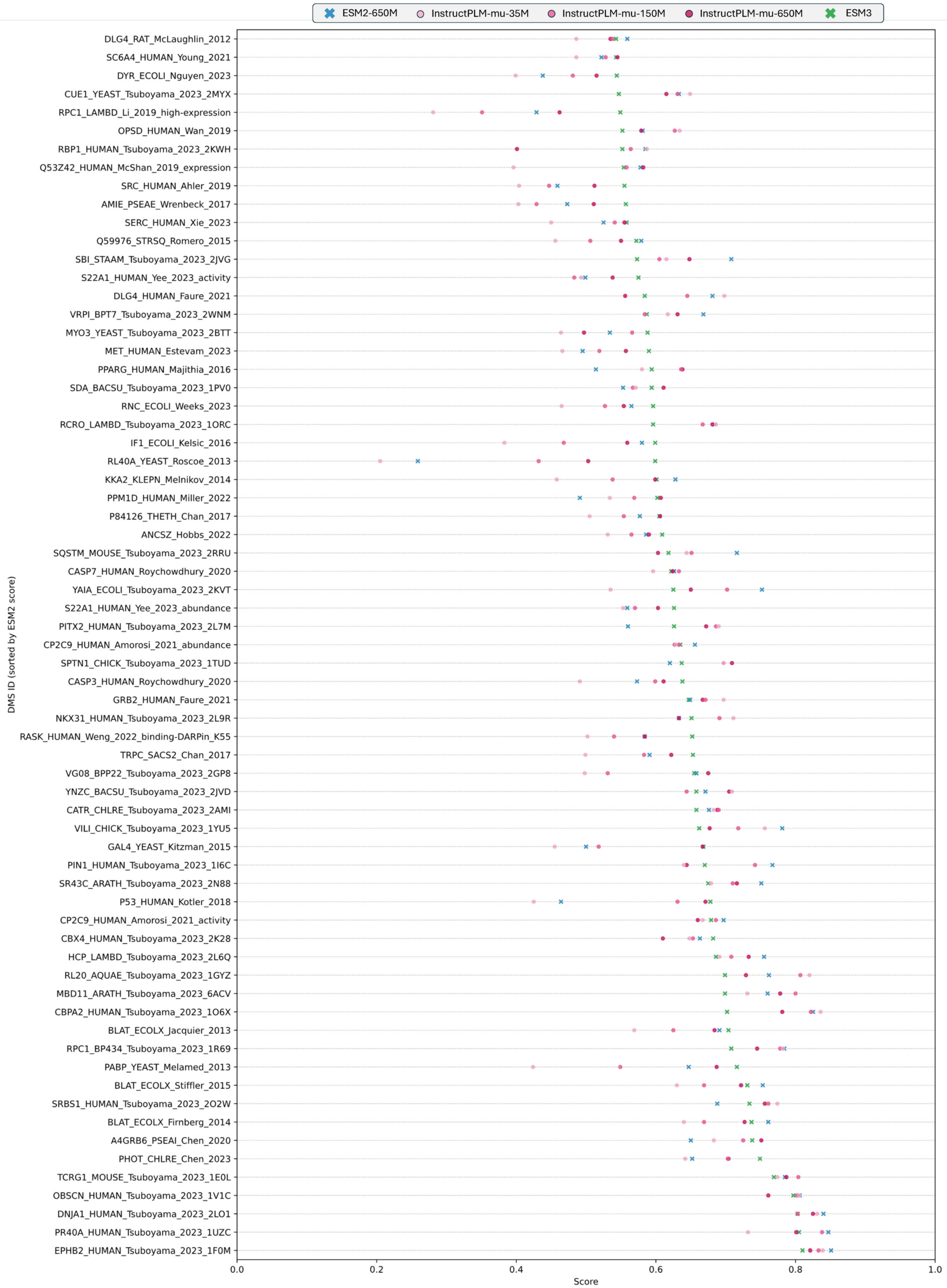

Figure S3 Spearman correlations on individual DMS datasets, sorted by ESM3 performance. Baselines use cross markers; InstructPLM-mu are shown as colored circles.
